# International COVID-19 mortality forecast visualization: covidcompare.io

**DOI:** 10.1093/jamiaopen/ooab113

**Published:** 2021-12-28

**Authors:** Samir Akre, Patrick Y Liu, Joseph R Friedman, Alex A T Bui

**Affiliations:** 1 Medical Informatics Home Area, University of California, Los Angeles, California, USA; 2 Department of Radiological Sciences, University of California, Los Angeles, California, USA; 3 Center for Social Medicine and Humanities, University of California, Los Angeles, California, USA

**Keywords:** COVID-19, mortality, data visualization, forecasting, global health

## Abstract

COVID-19 mortality forecasting models provide critical information about the trajectory of the pandemic, which is used by policymakers and public health officials to guide decision-making. However, thousands of published COVID-19 mortality forecasts now exist, many with their own unique methods, assumptions, format, and visualization. As a result, it is difficult to compare models and understand under which circumstances a model performs best. Here, we describe the construction and usability of covidcompare.io, a web tool built to compare numerous forecasts and offer insight into how each has performed over the course of the pandemic. From its launch in December 2020 to June 2021, we have seen 4600 unique visitors from 85 countries. A study conducted with public health professionals showed high usability overall as formally assessed using a Post-Study System Usability Questionnaire. We find that covidcompare.io is an impactful tool for the comparison of international COVID-19 mortality forecasting models.

## OBJECTIVES

Predicting the trajectory of COVID-19 mortality remains a matter of great international interest. Policymakers require timely and precise forecasts of likely scenarios to make informed decisions about preventive measures and resource implementation. In this context, dashboards to visualize COVID-19-related data have been shown to be impactful in the COVID-19 response.^1^ However, a search on PubMed for “covid-19 mortality forecast” returns over 2000 search results. The plethora of published mortality forecasting methods—where methods and predictions can vary drastically—make it a challenge to come to an informed choice about which model performs best, given different environments and conditions.

Monitoring and comparing different forecasting models is increasingly important, as disparities in COVID-19 vaccine allocation and consequent inequalities in disease burden have grown over the course of the pandemic. As of June 2021, data from Our World in Data show that Israel, the United Kingdom, and the United States have vaccination rates of 123, 110, and 96 doses per 100 people, respectively; in contrast, the Democratic Republic of Congo has less than 0.1 administered doses per 100.^2^ Thus, vaccine disparities are likely to dramatically alter how mortality forecasts perform in different countries. Further, in high-income contexts like the United States, differential vaccine hesitancy has led to pockets of very low COVID-transmission in some areas and exponential transmission in others. These gaps are of high concern as new, more highly transmissible variants of SARS-CoV-2 are spreading globally.^3^ As such, accurate, localized forecasts for mortality can aid in COVID-19 policy decision-making at the international, national, and local levels.

Several COVID-19-related visualization platforms existed prior to covidcompare.io and served as inspiration and starting points for this work.^4^^,^^5^ However, most of these visualizations focus on displaying results from a single modeling effort and were created without usability assessment. Moreover, each has established a distinct format, making comparisons difficult and time consuming for a policymaker interested in comparing diverging predicted trends. Markedly, the visualization tools that compare several models have been largely limited to the United States, such as the visualizations from FiveThirtyEight^6^ and the COVID-19 Forecast Hub.^5^

Friedman et al^7^ created a scientific framework for comparing the forecasts of different groups and institutions that predict COVID-19 mortality internationally. They identified 7 modeling groups with global mortality prediction data finding they had between 7% and 13% median absolute percent error (MAPE) in October 2020. These modeling groups frequently publish new versions of their model with updated predictions. By capturing each published iteration of these disparate models, the framework allows one to assess which model has performed best and how it has changed over the course of the pandemic. The analysis framework can be employed globally, at the level of individual countries; and for the United States, per individual state. This case report implements the framework from Friedman et al through a web-based data visualization creating a useable, up-to-date platform to compare COVID-19 mortality forecasts.

## MATERIALS AND METHODS

### Source data

Projected COVID-19 mortality from 6 international forecasting models, recorded ground truth mortality, and historical model errors are captured and preprocessed as described in Friedman et al.^7^ The data are uploaded to an SQL database used by our visualization tool, covidcompare.io.

### Tool development

The visualization tool is created using the Next.js framework for website development in React, a JavaScript-based library for user interface (UI) creation. Next.js allows both server-side rendering and static site generation for quick page load times. The website was built to work across a variety of screen sizes including mobile phones and tablets. Data visualizations were built using the d3.js-based Recharts library and react-grid-heatmap. The website was coded by SA and source code is available on GitHub (https://github.com/akre96/CovidCompare).

### Website layout and design

The landing page is aimed at allowing users to find up-to-date COVID-19 mortality forecasts for their chosen region. It displays a graph of the most recent COVID-19 death rate (deaths per day) predictions for a given country with a 7-day rolling average filter applied, along with the past 3 months of recorded mortality data. Models that actively update their forecasts are highlighted and may have their forecasts toggled on or off for the display. Users can choose to view cumulative mortality, expand the domain of the graph to show up to a year of historical mortality data, remove the rolling average filter, and/or show 95% prediction intervals for estimates where available.

Users can also access the site’s model performance page to compare historical performance of different modeling groups for a geographic region of interest. The model performance page displays a heatmap of MAPE. On the heatmap, the vertical axis shows the modeling group, and the horizontal axis shows the number of weeks out from model creation date. Viewers can select from various error metrics and time intervals (ie, cumulative or weekly errors and different month evaluation periods) within geographic areas. For a deeper look at a single modeling group, the historical forecast page allows a user to discover any systematic patterns of errors a given model has had for a specific country. Predictions from all versions of a model are displayed and can be filtered for models created within a specific range of months. This format allows users to investigate for patterns of error—such as underestimations—and adjust their confidence in a prediction accordingly.

### Example use case

**Figure 1.**
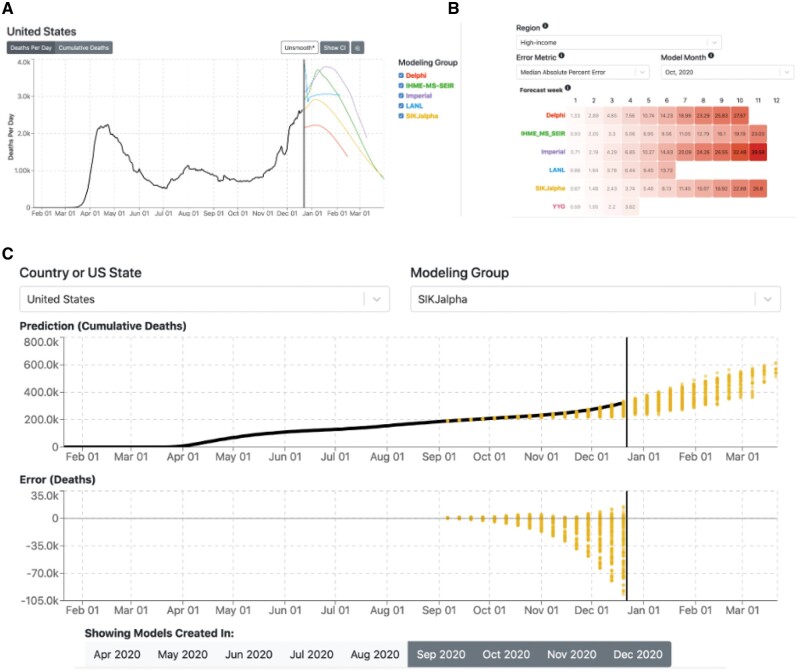
covidcompare.io visualization tool example images showing (A) current forecast page, (B) model performance page, and (C) historical forecasts page.

In mid-December 2020, a policymaker wants to know how many daily COVID-19 deaths the United States is likely to have by March 2021 (Figure 1A). Seeing fairly large differences in predictions, they investigate how well different models have performed historically and notice that the IHME and USC-SIkjalpha models have the most accurate long-term predictions in high-income countries (Figure 1B). Looking further, they then see that the USC-SIkjalpha model has been routinely underestimating mortality in forecasts for the United States over the past 4 months (Figure 1C). The policymaker now knows that the best performing model in their region forecasts declining COVID-19 mortality rates by March, but that the same model tends to underestimate mortality which suggests that in March mortality rates may not reduce as much as predicted without intervention. These data allow a policymaker to suggest appropriately scaled intervention to reduce mortality rates.

### Impact and usability testing

Three methods were used to assess the impact and usability of the visualization tool: (1) a feedback form linked in the site footer allows users to rate their experience and suggest improvements; (2) a privacy-preserving website analytics tool, Plausible.io, embedded on the website to track anonymous behavior such as bounce rate (users that only view the landing page), unique visitors, country of user, and what source a user came from (eg, Twitter, Google search, or direct URL input); and (3) a usability assessment conducted with 8 public health professionals to gather a more detailed understanding of how the target audience of public health professionals and policymakers use the site. The usability assessment used the Post-Study System Usability Questionnaire (PSSUQ) version 2, a validated 19-item instrument taken after performing a task using any system interface.^8^ PSSUQ questions use a 7-point Likert scale (1, strongly agree; 7, strongly disagree) and maps to 1 of 3 domains: system usefulness, information quality, and interface quality. Hence, lower scores on the PSSUQ indicate better performance. The results of the PSSUQ are compared to the mean values of 21 project as reported in Lewis.^9^ For this formal usability assessment, 8 weeks out from the date of interview and before administering the PSSUQ, participants were asked to take on the role of a public health advisor in Brazil and produce their best estimate of cumulative COVID-19 mortality. Interviews were conducted over recorded Zoom sessions. During the interview, user interaction with the website was also observed to assess areas of improvement for usability. The study was listed as institutional review board (IRB) exempt by the UCLA Office of Human Research Protection Program (IRB #21-000172).

## RESULTS

### Website use

From our launch in early December 2020 to June 2021, covidcompare.io has received >4600 unique visitors and 18.4k page views. The bounce rate is the percent of users who leave the website without any interaction; covidcompare.io has a bounce rate of 31%, indicating that most users who visit interact with the website. Most visitors were from the United States (54%), followed by Mexico (7%), the United Kingdom (6%), India (3%), Columbia (3%), and 79 other countries accounting for the remaining 27% of visitors. Forty-four percent of users visited via mobile devices, 26% by laptop, 25% by desktop, and 5% by tablet. Most our visitors reached the website via a direct link (2.7k) followed by 1.6k reaching us through Twitter. The remaining 370 visitors reached covidcompare.io through a mixture of paths, including Google searches, Facebook posts, and Instagram.

Our peak number of visitors per month was in December of 2020 with 2407, largely driven by the attention gained from initial Twitter posts. In recent months, covidcompare.io has seen increasing visits per month: 344 in March, 396 in April, and 504 in May 2021, suggesting organic growth in new visitors to the website.

### Usability study results

**Figure 2.**
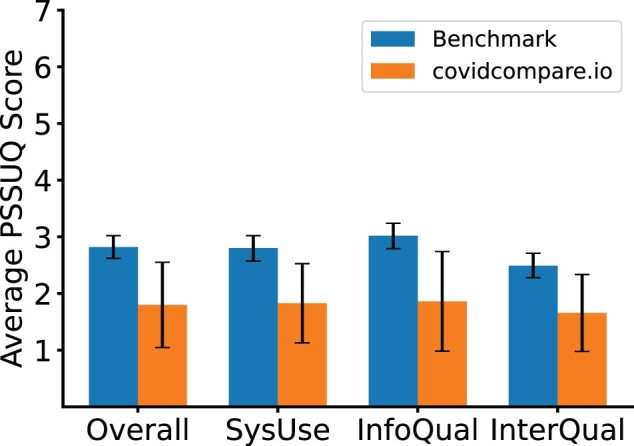
Performance of covidcompare.io (*n* = 8 participants) and benchmark comparison from 21 studies (*n* = 210 participants) on usability metrics. Scores shown for overall usability, system usability (SysUse), information quality (InfoQual), and interface quality (InterQual). Lower scores indicate better performance with a minimum possible value of 1 and a max of 7. Error bars indicate 99% confidence intervals.

The PSSUQ scores in the categories of system usability, information quality, and interface quality were significantly lower than a benchmark comparison from 21 studies with 210 participants total from Sauro and Lewis,^9^ indicating better usability of covidcompare.io as seen in Figure 2.

The direct observation of user interaction during the task preceding the PSSUQ revealed a key area for improvement. The region of interest (country or US state) being investigated is not stored across pages of the website, forcing users to reselect. When switching frequently between pages, the lack of session memory often led to users erroneously looking at the default country/region until realizing their error. No other issue was consistently noted across interviews. Overall, users indicated that they found the interface visually pleasing and intuitive to use.

## DISCUSSION

Covidcompare.io was built to be user friendly, fast, and effective. The results from tracking covidcompare.io usage via Plausible.io metrics indicate that covidcompare.io is being used with an increasing, international userbase and high engagement as indicated by the low bounce rate. From the usability study, we find that our target audience of public health professionals can effectively use the tool to answer questions of relevance to COVID-19 planning. These 2 findings indicate that covidcompare.io is an effective and engaging system to compare global COVID-19 mortality forecasting groups.

### Website development limitations

When designing a website geared at enabling in-depth analysis, it is critical to balance technical completeness with usability and accessibility. Throughout the development process, we attempted to balance the needs of most users (being able to see the forecasts in a region) while allowing for deeper study for those aiming to understand which models work best and when. The usability study helped to ensure that we adequately designed the website to work for the in-depth analysis user group but does not test if a general population would find the website usable.

## CONCLUSION

With covidcompare.io, one can analyze models given evolving world circumstances. This approach of building a visualization tool for a robust method of analysis enables the continued utility of scientific work and has potential to improve real-world application of work done across academic domains.

## FUNDING

SA receives funding from the National Institutes of Health (NIH) National Institute of Biomedical Imaging and Bioengineering (NIBIB) Medical Imaging Informatics Training Grant (T32 EB016640).

## AUTHOR CONTRIBUTIONS

SA built the covidcompare.io website, conducted the usability study, and wrote the manuscript. PYL provided data management for covidcompare.io, was involved in the design and implementation of the website, and reviewed and edited the manuscript. JRF was involved in the design and implementation of the website and reviewed and edited the manuscript. AATB advised and guided the project and reviewed and edited the manuscript.

## CONFLICT OF INTEREST STATEMENT

None declared.

## DATA AVAILABILITY STATEMENT

Usability study responses to the PSSUQ available at https://doi.org/10.5068/D1V68X.
